# Case report: A comprehensive report on the first confirmed Mpox case in the Philippines during the 2022 Mpox global outbreak: from clinical presentation to shotgun metagenomic sequencing analysis

**DOI:** 10.3389/fmed.2024.1387407

**Published:** 2024-10-22

**Authors:** Edward Matthew Ylaya, Phoebe Grace Grande, Lei Lanna Dancel, Amalea Dulcene Nicolasora, Francisco Gerardo Polotan, Roslind Anne Pantoni, Ezekiel Melo, Stephen Paul Ortia, Joanna Ina Manalo, Miguel Francisco Abulencia, Maria Yna Joyce Chu, Timothy John Dizon, Ma Carmela Bucoy-Sy, Gisella Adasa, Aileen Gianan-Gascon, Arthur Dessi Roman

**Affiliations:** Research Institute for Tropical Medicine, Muntinlupa, Philippines

**Keywords:** Mpox, dermatopathology, shotgun metagenomic sequencing, polymerase chain reaction, Philippines

## Abstract

We report a case of a 31-year-old Filipino male with travel history to several European countries in July 2022. He developed five non-tender, well-defined, umbilicated pustules with erythematous borders on the upper lip, left gluteal area, bilateral knees, and left ankle. Skin punch biopsy findings were suggestive of a viral infection. Mpox infection from Clade II (previously known as the West African clade) was confirmed by detecting and amplifying the G2R_G, G2R_WA and C3L gene targets using qPCR. Shotgun metagenomic sequencing subsequently identified a Mpox genome sequence belonging to B.1.3 lineage of Clade IIb, associated with the current multi-country outbreak. Serologic varicella IgM test was positive but varicella PCR of the skin lesion and metagenomic sequencing did not indicate the presence of the varicella virus. The patient was discharged and continued isolation at home until all scabs had completely fallen off. The presence of pustules among patients with risk factors such as possible close physical contact with infected individuals in areas with reported cases of Mpox should raise suspicion for such an infection. Establishment and optimization of qPCR protocol were necessary to confirm Mpox infection. Metagenomic sequencing successfully characterized the etiologic agent of the first laboratory-confirmed Mpox case in the Philippines belonging to Clade IIb which is mainly responsible for the 2022 Mpox global outbreak.

## 1 Introduction

Mpox, formerly known as monkeypox, is caused by the mpox virus (MPXV). It is historically endemic in African countries, hence the name of its two clades: clade I (formerly the Central African clade) and clade II (formerly the West African clade). Two subclades from clade II (subclades IIa and IIb) were identified when a multi-country Mpox outbreak happened in non-endemic countries in early 2022 (1). On July 23, 2022, the World Health Organization (WHO) declared Mpox as a Public Health Emergency of International Concern (PHEIC) (1, 2). At time of writing, there have been more than 98,000 reported cases worldwide with 94,623 cases found in locations that have not historically reported Mpox (3). A total of 183 deaths from Mpox have been recorded in more than 100 countries, including 162 deaths that occurred in non-endemic regions (3). The Mpox global outbreak last 2022 prompted further investigation on the possible linkage of the Mpox viral evolution to the geographic spread of disease.

Case detection of people with Mpox in non-endemic countries during the outbreak was challenging. In the Philippines, only one national reference laboratory, the Research Institute for Tropical Medicine (RITM), was capable of Mpox lab-based diagnosis during the outbreak. Real-time polymerase chain reaction (rt-PCR) was the standard diagnostic test for Mpox diagnosis in the Philippines. Shotgun metagenomic sequencing became useful in identifying genome sequences of the MPXV for phylogenetic characterization with the primary goal of determining the source of infection (4). In the absence of targeted whole genome sequencing protocols of acceptable performance during this time, this method have been widely used by laboratories worldwide to trace the common origin for the outbreak.

We report the first ever confirmed Mpox case in the Philippines, with a clinical presentation different from the classic Mpox cases that were previously described in endemic countries prior to the 2022 outbreak. We demonstrate how Mpox appears grossly on Southeast Asian brown skin by, describing the dermatopathological findings of the umbilicated pustule. We also discuss the use of Mpox rt-PCR for diagnostic confirmation, and the pioneering application of shotgun metagenomic sequencing to characterize the infecting virus.

## 2 Case presentation

**Figure 1:**
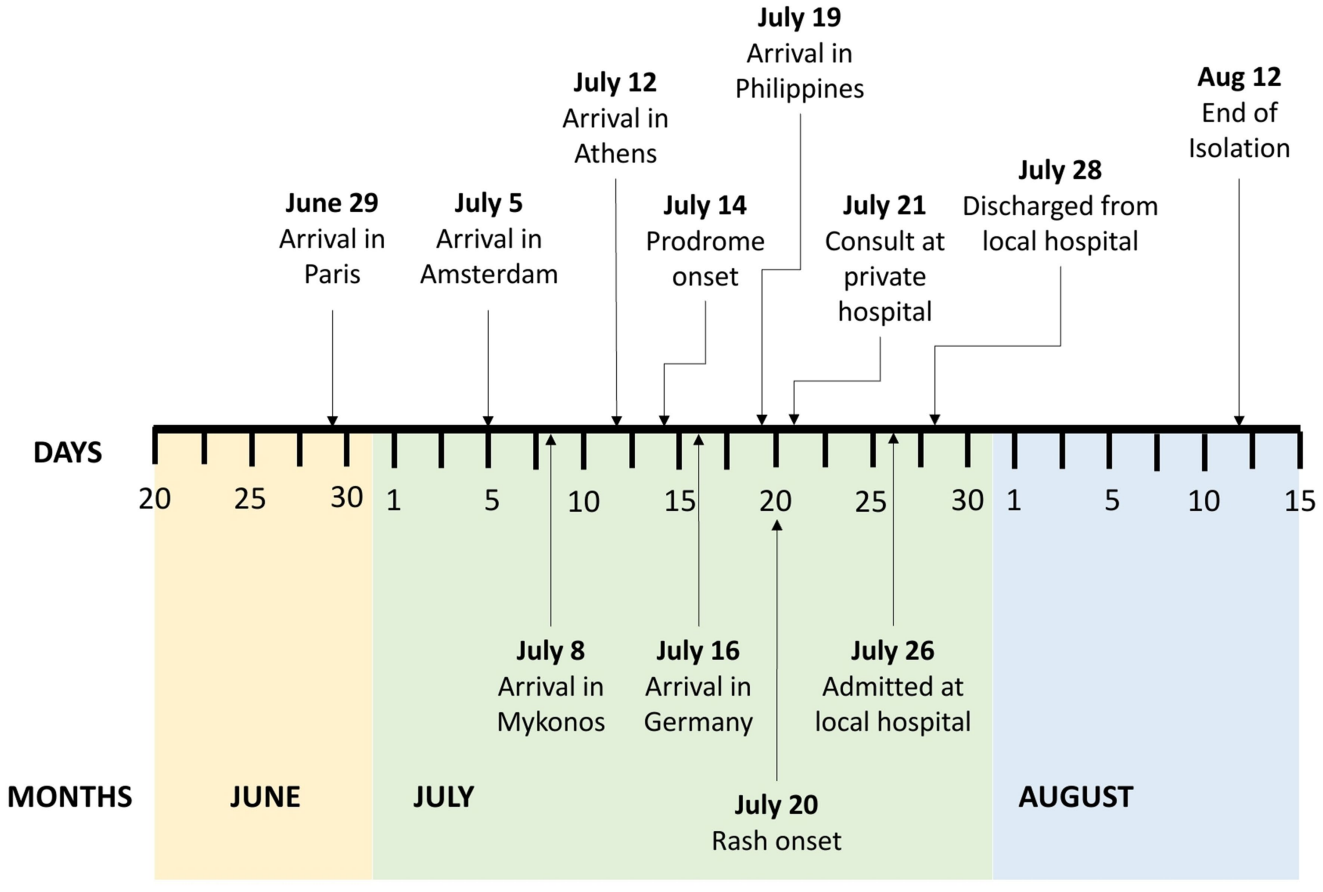
Timeline of patient activities and potential exposures to Mpox virus, June–August 2022.

A 31-year-old Filipino, male, homosexual, consulted last July 26, 2022 for pustules on his left ankle, left gluteal area, left upper lip, and both knees. Four weeks prior to consultation, he had a 3-week-long leisure trip to several European cities where he attended a concert and several social gatherings (Figure 1). He reported being unaware whether or not he had a close physical contact with Mpox confirmed nor suspect cases during these events. He also disclosed having interacted directly with stray animals at parks. He denied having any sexual activities while in Europe. He was apparently well until thirteen days prior to consultation when he had one episode of undocumented fever and chills, which resolved after taking a combined anti-inflammatory tablet. Seven days prior to consultation, he returned to the Philippines with no symptoms. But six days prior to consultation, pruritic vesicular rashes appeared on his left gluteal area, bilateral knees, and left anterior ankle. Five days prior to consultation, the patient reported having anal pain and bloody stools prompting visit at a nearby private hospital where he was sent home with prescribed diosmin + hesperidin tablets. Four days prior to consultation, he developed an ulcer on the right upper lip and had itchiness on the surface of his tongue. He also noted the increasing size of his knee lesions which urged him to consult at a local government hospital.

Upon further history taking, the patient had no known comorbidities, no known allergies to food or medications, and no previous Varicella, Smallpox, nor Measles infection. He cannot recall any vaccination history against Varicella, Measles, and Smallpox viruses. He was a nonsmoker, an occasional alcoholic beverage drinker, and denied illicit drug use. He only had one male sexual partner, who was asymptomatic at the time of consultation. He previously underwent human immunodeficiency virus (HIV) screening three months prior to consultation and had negative results. He also completed a course of HIV pre-exposure prophylaxis (PrEP; once-daily emtricitabine plus lamivudine combination tablet) two months prior to consultation, however he was not able to follow up after completion of the PrEP course.

**Figure 2:**
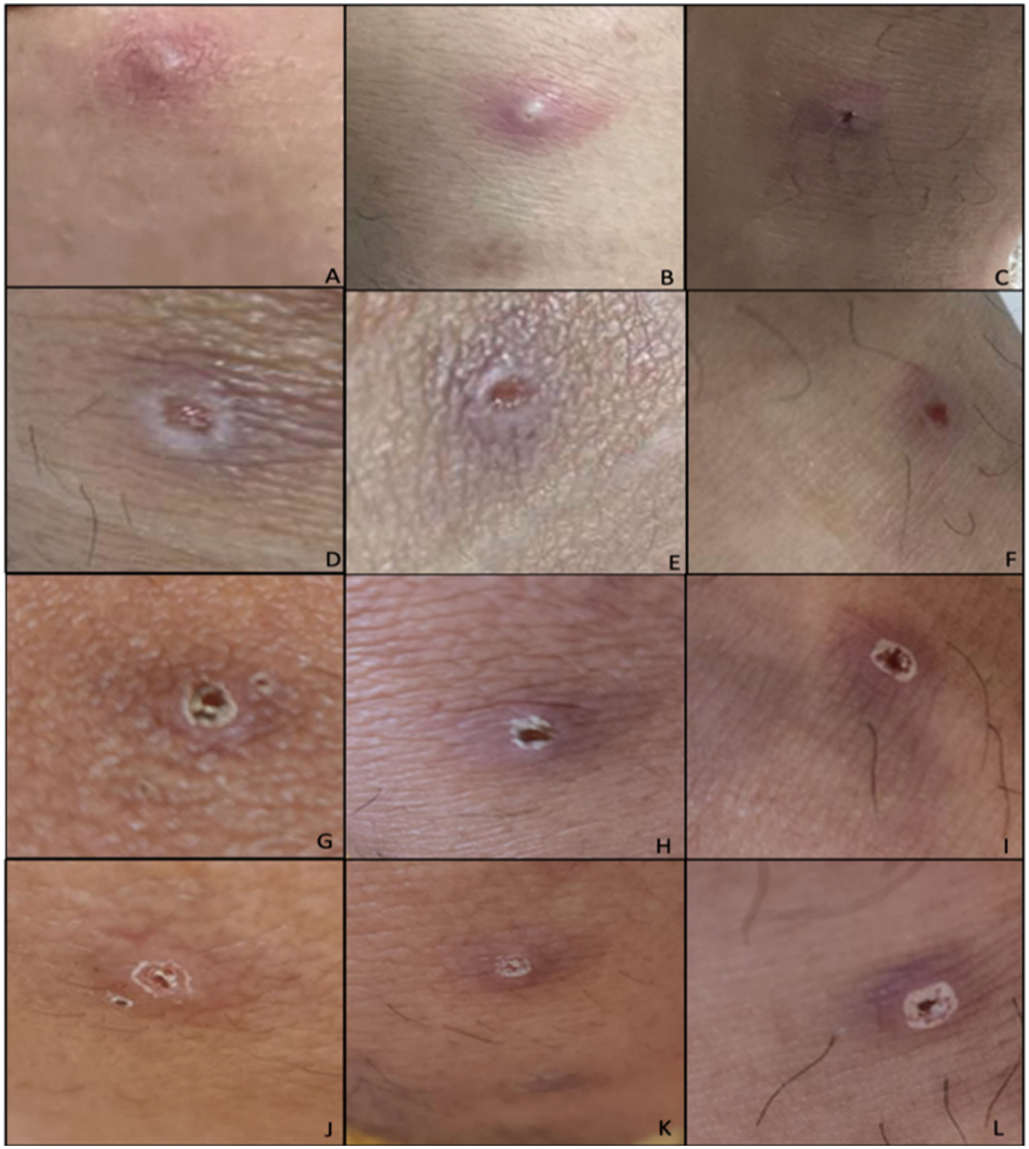
Initial lesions of the patient during admission (Days 6–12 from rash onset). Lesions located at: **(A)** Left knee, **(B)** right knee, **(C)** left ankle. Lesions after unroofing and swabbing on **(D)** left knee, **(E)** right knee, **(F)** left ankle. Lesions upon discharge from the hospital **(G)** left knee, **(H)** right knee, **(I)** left ankle. Day 12 of lesions on **(J)** left knee, **(K)** right knee, **(L)** left ankle.

On physical examination, the patient was seen awake, conscious, ambulatory, and with stable vital signs. Focused physical examination of the skin revealed five well defined, non-tender pustules with umbilication and erythematous borders on the right upper lip, left gluteal area, bilateral knees, and left ankle (Figure 2). No lymphadenopathies were noted. The patient was evaluated using the screening criteria for Mpox as follows: (1) presence of rash and any of the following: (2) associated with headache, fever, swollen lymph nodes, muscle and body pains, or weakness; (3) travel history to other countries, (4) history of contact with another person with rash, (5) history of prolonged or close physical contact with other individuals in the past 21 days, and (6) history of unprotected exposure to respiratory secretions or items used by confirmed cases of Mpox (5). After fulfilling three out of the six screening criteria for Mpox, the patient was tagged as an Mpox suspect hence admitted in an isolation room in the hospital for confirmatory testing and further evaluation.

Initial laboratory tests showed a normal white cell count of 8.6 × 10^9^/L, with 53% neutrophils and 35% lymphocytes. Serum creatinine, aspartate transferase (AST) and alanine aminotransferase (ALT) were all within normal limits. Other possible common infectious causes of rash in the patient were ruled out by the following test results: negative serologic measles Ag and Ab test, negative serum rapid plasma reagin (RPR) for syphilis, and negative herpes simplex virus (HSV-1 & 2) PCR of the skin lesions. Interestingly, the patient had a positive Varicella IgM result despite a negative Varicella PCR test of the patient’s skin lesions. The patient also had a negative HIV 1/2 antibody test. No testing for hepatitis B and C, Neisseria gonorrhea or chlamydia was done.

**Figure 3:**
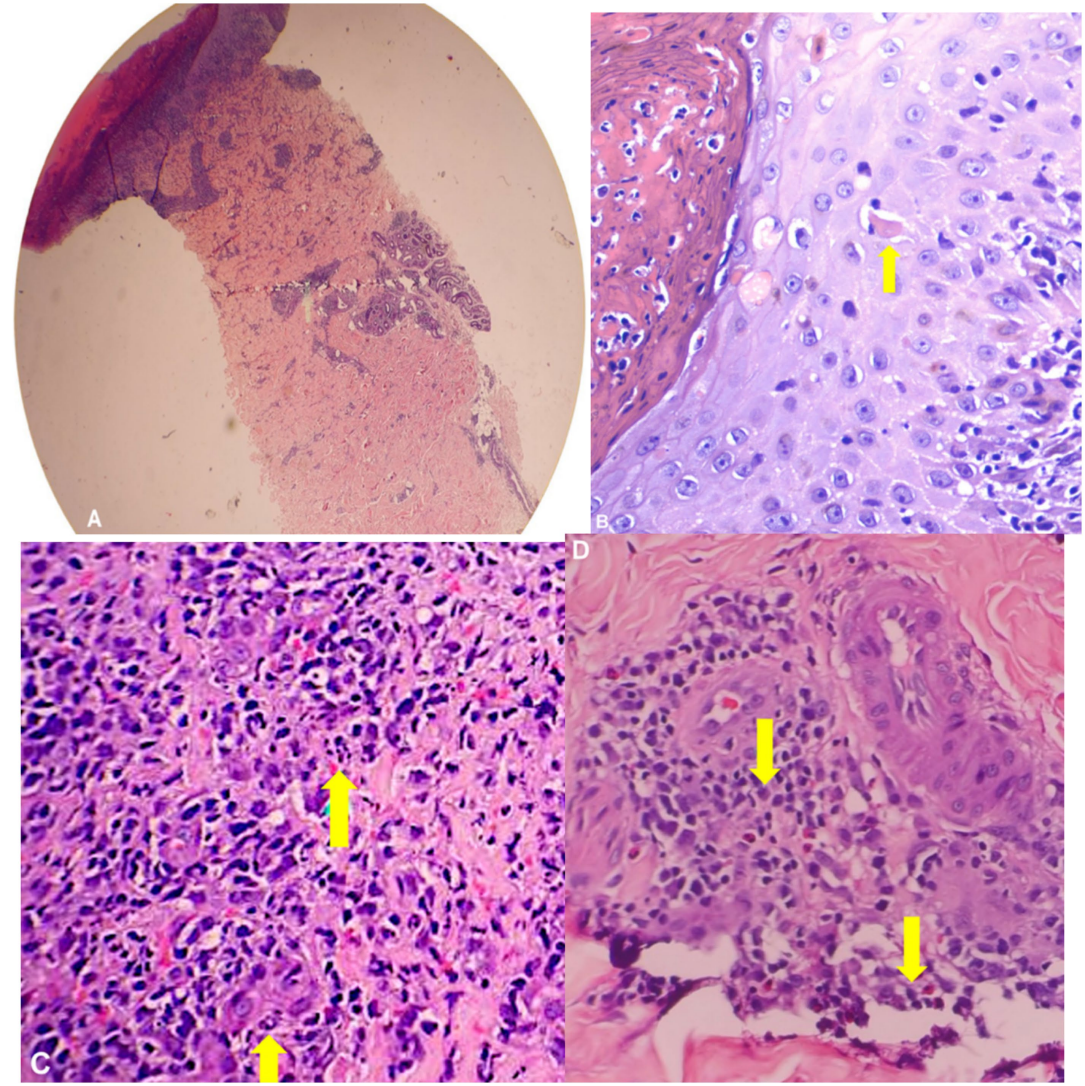
Skin punch biopsy (H&E). **(A)** On scanning view, section shows full thickness epidermal necrosis with **(B)** necrotic keratinocytes and a moderately dense, superficial and deep perivascular infiltrate of **(C)** neutrophils, lymphocytes, and **(D)** plasma cells.

The patient was also referred to the Dermatology service which performed skin punch biopsy. The skin punch biopsy results were consistent with the picture of a viral infection (Figure 3). The epidermis revealed scale crusts and ulceration. Some of the keratinocytes in the epidermis and the upper dermis were large with convoluted steel-gray nuclei and the dermis revealed red blood cell extravasation and a moderately dense, perivascular and interstitial inflammatory infiltrate of lymphocytes, histiocytes and plasma cells.

Mpox infection was considered given patient’s clinical and travel history, negative tests for other disease entities that are known to cause rashes, and skin punch biopsy suggesting a viral infection. The patient’s skin lesions were subjected to Mpox qPCR test and metagenomic sequencing. A total of nine tissue/lesion specimens and nine swab specimens obtained from three sites (right and left knees, and left ankle area) were sent to the Special Pathogens Laboratory for confirmatory Mpox rt-PCR test. The PCR primers and probes used were developed from the sequences described by Li et al. (6). Probe-based real time PCR assay was performed using Applied Biosystem’s AgPath-ID One Step PCR kit (4387424) (7) and Bio-Rad CFX96 Touch real time PCR machine as PCR platform. RNase P was the assays’ internal target control. The optimized primer and probe concentrations were 10 μM and 5 μM, respectively for both screening and differentiation assays (Supplementary Table S1). -ID One Step PCR kit (4387424) (7) and Bio-Rad CFX96 Touch real time PCR machine as PCR platform. RNase P was the assays’ internal target control. The optimized primer and probe concentrations were 10 μM and 5 μM, respectively for both screening and differentiation assays (Supplementary Table S1).

Two lesion dry swab specimens (right knee and left knee) and two lesion crust specimens (right knee and left ankle) were confirmed to be positive for Mpox viral DNA using rt-PCR screening assay with a mean cycle threshold (Ct) value of 21.24. Furthermore, the Mpox rt-PCR differentiation assay revealed that the same samples were positive for clade II only with mean Ct value of 20.79 (Supplementary Table S2).

To further characterize the etiologic agent of the first laboratory-confirmed Mpox case for the country, and properly classify its phylogenetic lineage, the four specimens that tested positive for Mpox rt-PCR were processed by the Molecular Biology Laboratory for metagenomic sequencing. Additionally, two confirmed target-negative samples were included in the sequencing run to be used as Mpox negative specimen control. The standard Illumina DNA Prep protocol was followed (8). The samples that passed the QC criteria were pooled and subjected to shotgun metagenomic sequencing using the Illumina Miseq sequencing instrument. Out of the four specimens that tested positive for Mpox real-time PCR, only three samples qualified for shotgun metagenomic sequencing.

The publicly available ‘Mpox-nf’ workflow developed by the Public Health Agency of Canada’s National Microbiology Laboratory (9) was adapted for generating Mpox consensus sequence. 10x and 5x depth thresholds and the MT903343.1 sequence from the B.1 hMPOX (human Mpox) lineage was used as the reference sequence for assembly. The script weeSAM was used to generate coverage depth plots (10). Nextclade was used for clade and lineage assignment, identification of single nucleotide variant (SNV) mutations, insertions, deletions, and for phylogenetic placement of sequences on a reference tree. Tablet was used to inspect aligned reads supporting the identified SNV mutations.

Supplementary Table S3 and Supplementary Figure S1 show the number of reads, sequencing depth, and genome coverage generated by the three Mpox positive samples. The total number of reads for each specimen ranges from 0.8 M to ~1.2 M reads wherein around 2.8 k to 7.7 k were mapped to the Mpox reference sequence (MT903343.1). Since all three samples were collected from the same patient, sequences generated by the three specimens were pooled to increase the number of reads and genome coverage. A total of ~5 M reads with 14.5 k reads mapped to the Mpox reference sequence and only 15% genome coverage using 10x default Illumina depth threshold. The genome coverage was increased to 80% when the depth threshold was lowered to 5x however this increased the likelihood of misclassifying mutations.

The Nextclade analysis of consensus sequences generated from all three separate MPOX22-0034 samples, the pooled MPOX22-0034 sample, and using 10× and 5× depth thresholds with respect to a reference sequence from the hMPOX outbreak clade (i.e., MPOX_USA_2022_MA001 in NC_063383 coordinates or pseudo_ON563414) is shown in Supplementary Figure S2. Only up to three single-nucleotide variant (SNV) mutations were identified when comparing the consensus sequences to this reference sequence, which was from a sample collected in May 2022.

**Figure 4:**
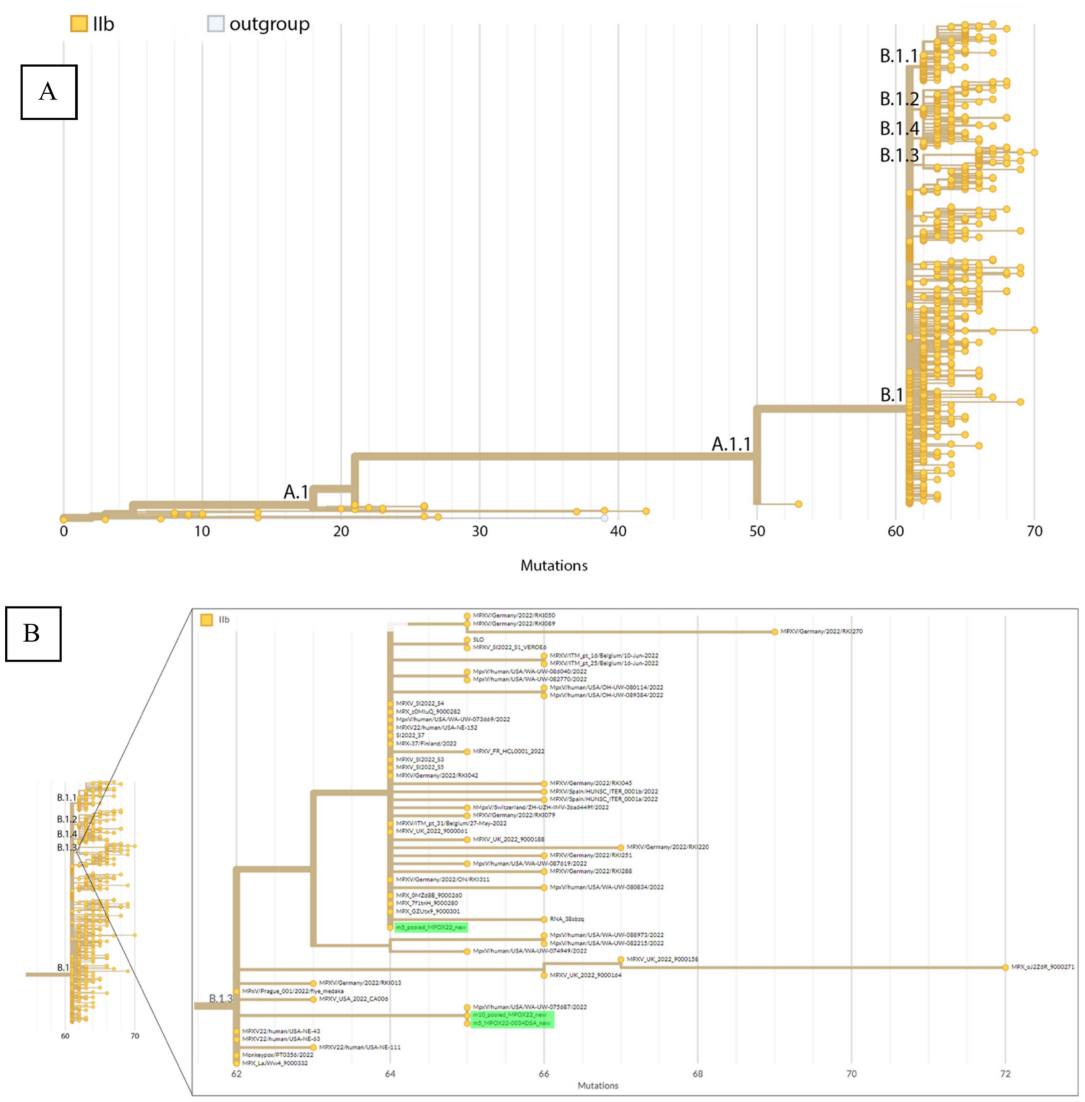
Phylogenetic placement by Nextclade of the Mpox consensus sequences. **(A)** Phylogeny representing the different hMPOX lineages including A, B, and their sublineages. **(B)** Zoomed-in view of the B.1.3 sublineage with red arrows indicating consensus sequences from sample MPOX22-0034 placed within this sublineage.

Figure 4 shows the phylogenetic placement by Nextclade of the Mpox consensus sequences on a reference tree representing the different lineages under the hMPOX clade. The consensus sequences are placed in the B.1/B.1.3 lineage, showing that the first detected Mpox case in the Philippines belongs to the B.1 or more specifically the B.1.3 lineage. Sequences with lower % coverage (MPOX22-0034DSA with 5× depth threshold and pooled_MPOX22 with 10× depth threshold) are placed at the base of the B.1.3 lineage while the sequence with the highest % coverage (pooled_MPOX22 with 5× depth threshold) is placed in a subtree within the B.1.3 lineage wherein the consensus sequence clusters with sequences from multiple European countries (including France, Germany, Finland, Switzerland, Spain, Belgium, and Slovenia) and the United States.

The three detected SNV mutations from the pooled_MPOX22-0034 consensus sequence are supported by 100% of reads covering their respective genome positions, which are the coordinates 55,133, 64,426, and 190,660 in the pseudo_ON563414 reference sequence (for more details, see Supplementary Figure S3). False positive mutations among these three SNVs are thus unlikely. Two of these mutations (G55133A/OPG074:R665C and C64426T/no amino acid change) are unique to B.1.3 (Supplementary Figures S3A,B), supporting the validity of the lineage assignment of the consensus sequence. C64426T is unique to the cluster of B.1.3 sequences originating from multiple European countries. One of the three mutations (G190660A/NBT03_gp174:R84K) appears in both B.1.3 and B.1 sequences.

Since the genome recovered from the sample clustered within a subtree of the B.1.3 lineage and the patient traveled to European countries where B.1.3 was circulating, the authors attempted to reconstruct ancestral states and determine the specific country that was the source of the infection. We used a common method used during the SARS-CoV2 pandemic, the Ultrafast Sample placement on Existing tRees (UShER), to find the placement of our MPXV sequence in a global phylogenetic tree (11). The result showed that many international sequences are identical with the first confirmed Mpox case sequence in our study in terms of mutations, because Mpox as a DNA virus accumulates mutations at a much slower rate compared to an RNA virus like SARS-2, for example (Supplementary Figure S4). This makes it less feasible to resolve the origin country that introduced this strain to the Philippines using sequence data alone.

Meanwhile, because of the positive varicella IgM result, metagenomic sequence data were reviewed to verify if there is a co-infection. No sequence data indicated varicella was present in the patient’s sample which supports the finding that there exists no true co-infection.

**Figure 5:**
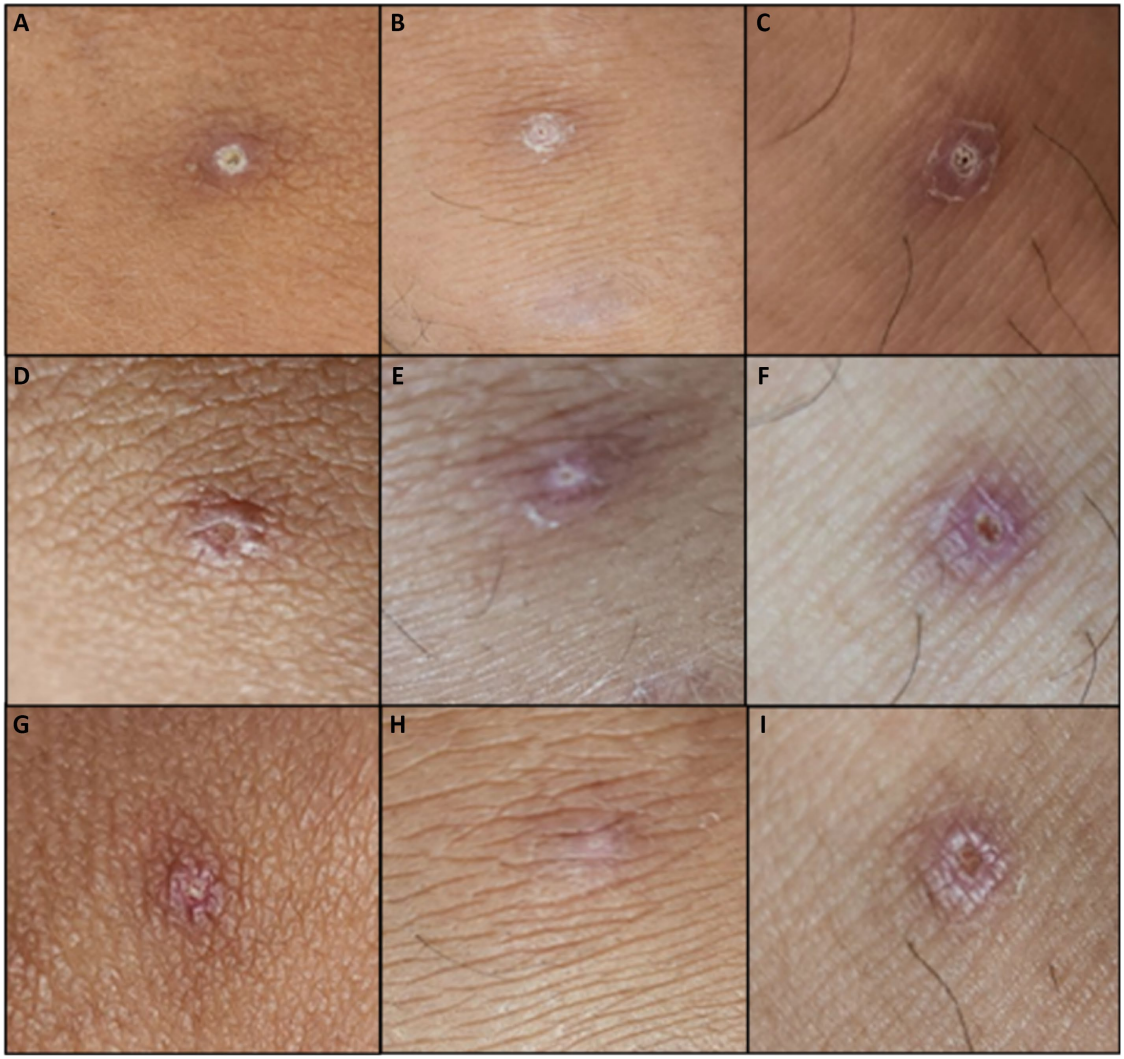
Lesions during home isolation (Days 17–23 from rash onset). Day 17 of lesions on **(A)** left knee, **(B)** right knee, **(C)** left ankle. Day 21 of lesions on **(D)** left knee, **(E)** right knee, **(F)** left ankle. Lesions upon the end of isolation **(G)** left knee, **(H)** right knee, **(I)** left ankle.

After establishing the presence of Mpox infection in the patient with the aid of rt-PCR and sequencing, he was discharged on the second hospital day. Home isolation was advised for the patient. He was advised to keep the lesions clean and dry, with the use of mild soap and moisturizing lotion daily as supportive management. Daily monitoring of the patient’s symptoms through online consultation with an infectious disease physician was done until all crusts and scabs had completely disappeared from the patient’s skin (Figure 5). The prognosis of the patient’s illness was good. No complications nor worsening of symptoms were reported by the patient during the isolation period which lasted for 23 days from rash onset or 15 days post-hospital discharge (Figure 1). Local contact tracing of the patient’s close contacts in the Philippines were done and no identified close contact became symptomatic nor developed any rash. Unfortunately, contact tracing of the patient’s possible close contacts in the European cities that he visited was not done.

## 3 Discussion

This report presented the first confirmed imported and travel-related case of Mpox virus in the Philippines in a patient with a three-week travel history to several European countries and clinical symptoms of few umbilicated pustules and a mild prodromal stage.

In countries where no Mpox cases have been reported historically, it is important to have epidemiological and clinical knowledge of Mpox infection for proper diagnosis and treatment. The classic form of Mpox reported from endemic areas had been known to have three stages: an incubation period of approximately 6–21 days; a prodromal phase characterized by the presence of fever, severe headache, lymphadenopathy, malaise, myalgia, and fatigue; and a rash period when the patient shows the typical rash progression (initially macular to papular/pustular lesions progressing to vesicular then umbilicated and later on becomes crusted), appearing more commonly on the face and limbs (12, 13). The Mpox case presented in this report went through all these three stages but his symptoms were considered “atypical” due to the presence of fewer than 10 skin lesions, with oral and anogenital involvement, and with rectal pain and/or bleeding (14–16). The source of the patient’s oral and anogenital skin lesions remains unknown. Although the patient denied any sexual activities during his European trip, it is still important to note that there were Mpox cases involving MSM (men having sex with men) during the Mpox outbreak in Europe who reported to have perianal and genital lesions. It was found that these lesions were mostly acquired through direct skin-to-skin contact during sexual intercourse (17). On the other hand, while hemorrhoids might be the cause of the patient’s rectal bleeding, it is possible that other less obvious mucosal lesions or Mpox-associated proctitis might have contributed to the rectal pain and bleeding as is reported in other case series (13, 15).

Fortunately, the Mpox real-time quantitative PCR assay has been optimized and set-up more than four weeks earlier than the patient’s consultation in our institute (18). This highlights the importance of increased disease information for early case detection, and timely establishment of national diagnostic capacity for confirmation of infection for emerging diseases as part of a comprehensive, multisectoral response plan. PCR remains to be the gold standard in diagnosing MPOX infections. The Ct values generated by the Mpox positive samples from the patient were low (Ct value range of 20 to 21) which denotes the presence of a high amount of Mpox viral RNA in the sample; Also, based on the analytical sensitivity test, nucleic acid extract concentrations from the lesion crust and dry swab specimens (4–12 ng/uL) were also found to be within the verified limit of detection for the G2R_G assay (2.92 × 100 copies/mL) and the G2R_WA assay (4.31 × 100 copies/mL). The PCR findings highlight the significance of the viral transmission of Mpox via direct contact with infected skin lesions. Hence, it is highly likely that the patient acquired the infection by skin-to-skin contact with another infected individual or animal.

The virus was identified to belong to hMPOX Clade II, formerly known as the West African Clade, and its sequence was placed in the B.1.3 lineage. Undetected mutations remain a potential limitation but, if present, are not likely to change the assigned lineage given the presence of B.1.3 unique mutations. This limitation can be resolved by sequencing with higher coverage depth. The nomenclature we used for describing the phylogenetic lineage is already consistent with WHO’s announcement last August 2022 referring to Clade I as the former Congo Basin clade and Clade II as the former West African clade. The primary basis for the new clade classification is the differences in the coding regions of each clade that relates to the immunomodulatory and host recognition antigenic determinants such as H3L and B12R (19). Clade II is now divided into two subclades Clade IIa and Clade IIb, the latter being the cause of the 2022 multi-country Mpox outbreak and infecting our patient after traveling to Clade IIb-reporting countries in Europe (20).

Mpox and varicella co-infection have been reported in the Democratic Republic of Congo (21). Such co-infection can occur when an initial infection facilitates entry of the second through breaks in the skin or when a first virus weakens the immune system increasing the susceptibility to a secondary infection (21). The positive varicella IgM result in our case could likewise indicate an Mpox-varicella co-infection; however, a false positive varicella serology result is also likely. The patient had no history of vesicles appearing in crops at various stages of evolution consistent with a Varicella infection. Additionally, contact tracing was done which identified close contacts not having any prior or subsequent vesicular lesions in the succeeding 21 days. With no varicella detection from PCR testing and metagenomic sequencing, we believe that a false positive serologic test is more likely. Previous studies have reported that IgM serologic testing is less sensitive and specific than Varicella PCR test of the skin lesions (22). At least three reasons have been identified: the presence of cross-reacting antibodies, interference by other existing autoimmune conditions that produce broadly specific heterophile IgM antibodies, and the increased false positives when a test is conducted in a low disease prevalence setting, similar to Mpox in the Philippines (23).

Currently, there are no available definite treatments for Mpox. There are also no drugs or vaccines against Mpox approved by the Philippine Food and Drug Administration as of the writing of this manuscript (5). Management is supportive and focused on symptom management and prevention of superinfection (5). Current local guidelines advise strict isolation of Mpox-confirmed cases until all symptoms have resolved. The patient reported in this case was advised to undergo isolation until all the scabs were gone to avoid further transmission, which lasted for 23 days. No serious complications that required further hospitalization were reported by the patient during his home isolation. He did not receive any antibiotics or antivirals.

## 4 Conclusion

The first laboratory-confirmed case of Mpox in the Philippines during the 2022 Mpox global outbreak presented with few, umbilicated papules in a young male with travel history to areas with active Mpox transmission. Biopsy of the lesions showed non-specific findings of necrotic keratinocytes with perivascular and interstitial inflammatory infiltrate of lymphocytes, histiocytes and plasma cells. An optimized qPCR protocol followed by the pioneering use of shotgun metagenomic sequencing confirmed infection with Mpox Clade IIb, more specifically, to the B.1.3 lineage associated with the 2022 Mpox outbreak in different European countries. The negative varicella PCR of the skin lesions supported by the absence of varicella DNA on the sequencing makes the varicella serologic IgM results as more likely false positive. This well-documented experience with Mpox will provide a model for subsequent timely clinical case recognition and laboratory confirmation for non-endemic, but high-risk country like the Philippines.

## Data Availability

The datasets presented in this study can be found in GenBank with accession number: PP415529 (BankIt2801701 hMpxV/Philippines/RITM-001/2022).
