# Supplementary material for: Case report: A comprehensive report on the first confirmed Mpox case in the Philippines during the 2022 Mpox global outbreak: from clinical presentation to shotgun metagenomic sequencing analysis
Source: Front Med (Lausanne). 2024 Oct 22;11:1387407. doi: 10.3389/fmed.2024.1387407 (PMC11541813; doi:10.3389/fmed.2024.1387407)
Supplement: Supplementary file 1 [file Data_Sheet_1.pdf]

A Comprehensive Report on the First Confirmed Mpox Case in the Philippines during the 2022 Mpox Global Outbreak: From Clinical Presentation to Shotgun Metagenomic Sequencing Analysis (Supplementary File)

Edward Matthew Ylaya[1†], Phoebe Grace Grande[1†], Lei Lanna Dancel[1], Amalea Dulcene Nicolasora[1], Francisco Gerardo Polotan[1], Roslind Anne Pantoni[1], Ezekiel Melo[1], Stephen Paul Ortia[1], Joanna Ina Manalo[1], Miguel Francisco Abulencia[1], Maria Yna Joyce Chu[1], Timothy John Dizon[1], Ma. Carmela Bucoy-Sy[1], Gisella Adasa[1], Aileen Gianan-Gascon[1], Arthur Dessi Roman[1]

[1]Research Institute for Tropical Medicine, Muntinlupa City, Philippines
[†]First authorship: These authors share first authorship

* **Correspondence to**:
Phoebe Grace B. Grande
phoebe.grande@ritm.gov.ph

A) B) C) D)

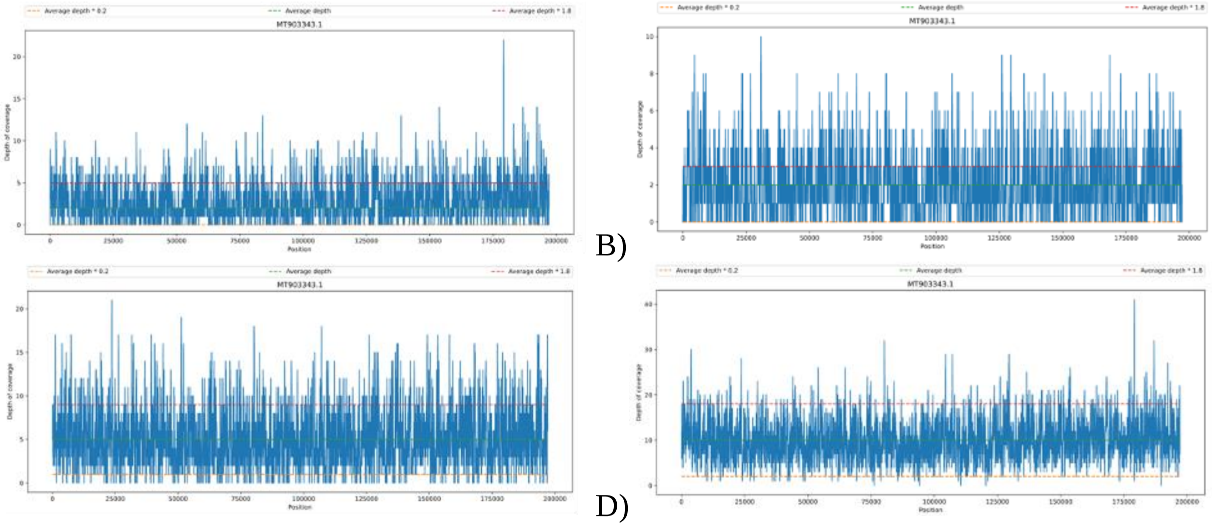

Figure S1. Coverage depth plots of reads aligned to the reference sequence MT903343.1. From samples (A) MPOX22-0034CH, (B) MPOX22-0034DSA, (C) MPOX22-0034DSE. and (D) pooled_MPOX22-0034. Figures 4a to 4c show wide coverage breadth over the whole MPXV genome but low coverage depth in all three separate samples between 2x to 5x. Coverage depth increased to an average of 10x in the pooled_MPOX22-0034 sample.

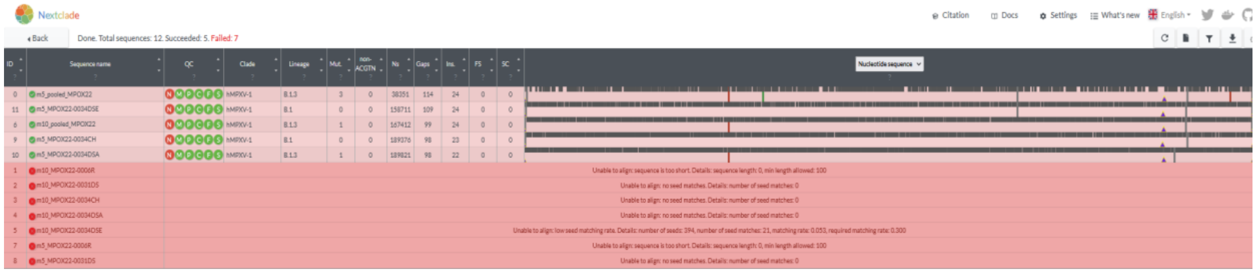

Figure S2. Nextclade Analysis of Consensus Sequences. From all three separate MPOX22-0034 samples, the pooled MPOX22-0034 sample, and using 10× and 5× depth thresholds with a reference sequence from the hMPXV outbreak clade

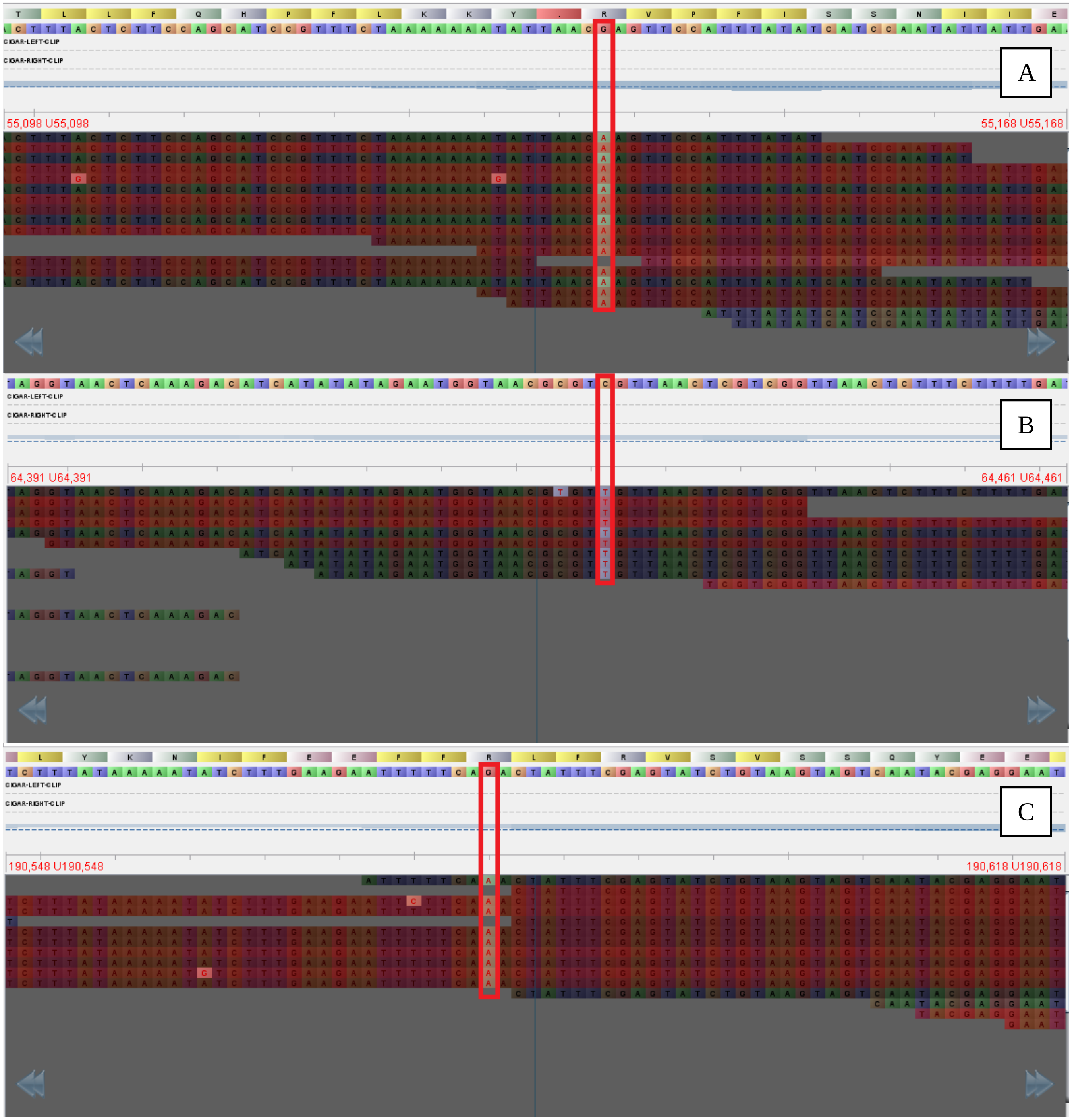

Figure S3. The three detected SNV mutations from the pooled_MPOX22-0034 consensus. Coordinates (A) 55133, (B) 64426, and (C) 190660 in the pseudo_ON563414 reference sequence.

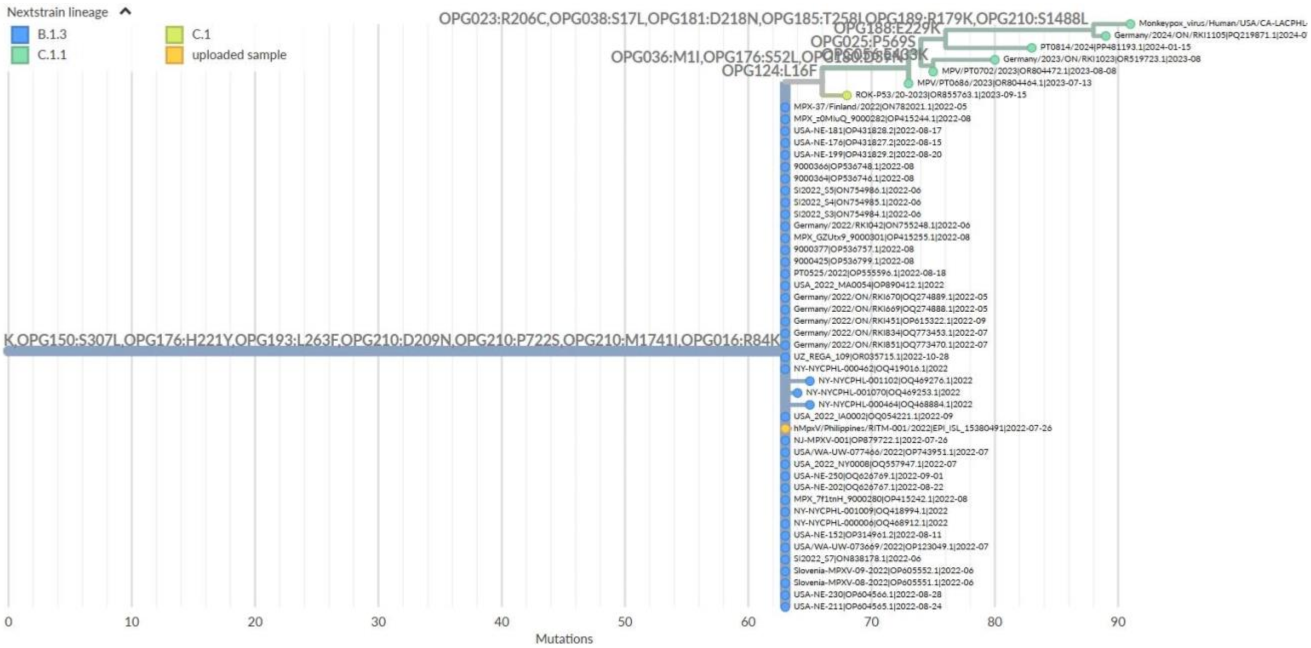

Figure S4. Ultrafast Sample placement on Existing tRee (UShER). Representative image of UShER result where the MPXV sequence of the first Philippine Mpox case is shown in the yellow dot, while the other sequences identical with it are seen on the long vertical line from the set of all available public MPXV sequences (23).

Table 1S. Primers and probes used in Mpox quantitative PCR test (5)

Primer/ Probe Name	Sequence 5’ to 3’	Assay Name
G2R_G Forward Primer	5'- GGAAAATGTAAAGACAACGAATACAG-3'	G2R_G (Mpox Screening)
G2R_G Reverse Primer	5'- GCTATCACATAATCTGGAAGCGTA-3'	
G2R_G Probe	5' FAM-AAGCCGTAATCTATGTTGTCTATCGTGTCC-3' BHQ1	
G2R_WA Forward Primer	5'- CACACCGTCTCTTCCACAGA-3'	G2R_WA (Mpox Differentiation)
G2R_WA Reverse Primer	5'- GATACAGGTTAATTTCCACATCG-3'	
G2R_WA Probe	FAM 5'-AACCCGTCGTAACCAGCAATACATTT-3' BHQ1	
C3L Forward Primer	5'- TGTCTACCTGGATACAGAAAGCAA-3'	C3L (Mpox Differentiation)
C3L Reverse Primer	5'- GGCATCTCCGTTTAATACATTGAT-3'	
C3L Probe	5' FAM-CCCATATATGCTAAATGTACCGGTACCGGA-3' BHQ1	

Table 2S. Mpox RT-PCR result with mean cycle threshold (Ct) values

Target	Sample	CT Value	Mean CT Value	Result
G2R_G	Lesion Base Dry Swab Right Knee	22.3	21.24	Mpox Viral DNA Detected
	Lesion Base Dry Swab Left Knee	20.57		
	Lesion Crust Right knee	19.57		
	Lesion Crust Left Ankle	22.52		
G2R_WA	Lesion Base Dry Swab Right Knee	21.86	20.79	Mpox Clade II** Viral DNA Detected
	Lesion Base Dry Swab Left Knee	20.14		
	Lesion Crust Right knee	19.06		
	Lesion Crust Left Ankle	22.08		
C3L	Lesion Base Dry Swab Right Knee	-	-	Mpox Clade I* Viral DNA Not Detected
	Lesion Base Dry Swab Left Knee	-		
	Lesion Crust Right knee	-		
	Lesion Crust Left Ankle	-		

**formerly classified as Congo Basin clade*

***formerly classified as West African clade*

Table 3S. Sequencing reads and MPOX genome coverage generated from the performed shotgun metagenomic sequencing. Assembly method: reference-based assembly with Mpox-nf workflow (bwa, samtools, ivar)

Sample	No. of paired-end reads from sample	No. of reads mapped to MPOX reference	Mean sequencing depth	Depth threshold (default for illumina=10x)	# Ns in consensus	Coverage over MPOX genome (0.0-1.0)
MPOX22-0034CH	1,210,779	3987	2.82958x	10x	197,134	0
				5x	189,399	0.0392373
MPOX22-0034DSA	829,239	2824	2.10975x	10x	197,134	0
				5x	189,831	0.037041
MPOX22-0034DSE	1,247,310	7721	5.1794x	10x	196,808	0.0016537
				5x	158,724	0.194801
Pooled MPOX22-0034	5,402,912	14,532	10.1189x	10x	167,425	0.150705
				5x	38,363	0.805382
